# Carbapenem-Resistant *Klebsiella pneumoniae*: Carbapenemase Production, Antibiotic Resistance and Treatment Options, in an Infectious Diseases Hospital from Romania

**DOI:** 10.3390/antibiotics15060533

**Published:** 2026-05-24

**Authors:** Alexandra Cireșă, Gabriel-Adrian Popescu, Daniela Tălăpan, Mihai Octavian Dan, Cristina Popescu

**Affiliations:** 1 Faculty of Medicine, “Carol Davila” University of Medicine and Pharmacy, 050474 Bucharest, Romania; 2 “Prof. Dr. Matei Balș” National Institute of Infectious Diseases, 021105 Bucharest, Romania

**Keywords:** NDM+OXA-48-producing *Klebsiella pneumoniae*, antibiotic synergy testing, ceftazidime–avibactam plus aztreonam efficacy

## Abstract

**Background/Objectives:** Carbapenem-resistant *Klebsiella pneumoniae* (CRKP) is of great concern because of the difficulties encountered in the management of infections it may cause. This study aims to identify possible difficulties in the management of *K. pneumoniae* infections in the current context of antibiotic resistance, particularly regarding carbapenem resistance. **Methods:** This is a retrospective, cross-sectional study that analyses epidemiological, clinical and bacteriological features identified in all patients with CRKP infections/colonization admitted during 2024 in an infectious diseases hospital. **Results:** Carbapenemase-producing *K. pneumoniae* isolates were co-harboring NDM+OXA-48 in 55.2% of cases. NDM+OXA-48-producing *K. pneumoniae* (116 isolates, 55.2%) was correlated with high resistance to aztreonam (100%, *p* = 0.01), ceftazidime–avibactam (100%, *p* < 0.01), trimethoprim–sulfamethoxazole (99.1%, *p* < 0.01), gentamycin (94.8%, *p* < 0.01), amikacin (93.8%, *p* < 0.01), colistin (79.8%, *p* < 0.01). OXA-48-producing *K. pneumoniae* (29 isolates, 13.8%) was correlated with lower resistance to ceftazidime–avibactam (11.5%, *p* < 0.01), amikacin (48.1%, *p* < 0.01), colistin (51.7%, *p* = 0.01), and gentamycin (65.5%, *p* < 0.01). We found in vitro synergistic effects of ceftazidime/avibactam + aztreonam for 32/32 CRKP isolates and of colistin + tigecycline for 12/14 CRKP isolates. Higher recurrence of CRKP infections was recorded in patients with urinary tract conditions (RR = 11.58, 95%CI: 1.58–81.91) and upper urinary tract devices (RR = 3.53, 95% CI: 1.72–7.22). In this study, adequate antibiotic treatment, compared to excessive antibiotic treatment in CRKP infections, was associated with shorter treatment duration (*p* = 0.02) and shorter length of hospitalization (*p* = 0.04). **Conclusions:** In our study, CRKP is frequently coharboring NDM+OXA-48, having limited treatment options. Implementing new treatment strategies, testing antibiotic synergies for older antibiotics in order to identify alternative treatment options and avoiding unnecessary carbapenem consumption are essential for decreasing the burden of CRKP infections.

## 1. Introduction

Carbapenems have been introduced in clinical practice as a major resource in the management of multidrug-resistant Gram-negative bacterial infections. In certain regions of the world, their extensive use, alongside the dissemination of carbapenem-resistant Gram-negative bacteria (CRGNB), led to the emergence of carbapenem resistance in roughly a decade after their introduction in clinical use [1]. *Klebsiella pneumoniae* is one of the bacterial species with an enhanced ability to develop and acquire antibiotic resistance, currently confronting elevated levels of carbapenem resistance in different areas of the world. Carbapenem resistance is commonly caused by carbapenemase production and less frequently by other mechanisms, such as porin loss or efflux pumps [2]. Carbapenemases, alongside all other beta-lactamases, are classified by structure into four classes under the Ambler classification system [3]. Carbapenemases commonly produced by *K. pneumoniae* belong to class A of serine-beta-lactamases (*K. pneumoniae* carbapenemase—KPC), class B of metallo-beta-lactamases (MBLs) (New Delhi metallo-beta-lactamase—NDM, Verona integron-encoded metallo-beta-lactamase—VIM, imipenemase—IMP) and class D of oxacillinases (OXA-48 and OXA-48-like) [3].

The first description of carbapenemases was 30 years ago, when the United States reported the identification of KPC (1996) [4]. Later, OXA-48 was first described in Turkey in 2001 [5], and NDM was first reported in Sweden in 2008, in a patient originating from India, with a history of treatment in a hospital from New Delhi [6]. In Europe, the emergence of carbapenem resistance prompted the launch of studies to analyze its evolution. In 2013–2014, the European Survey on Carbapenemase-Producing *Enterobacteriaceae* (EuSCAPE) identified KPC as the most frequent carbapenemase in *K. pneumoniae* (45%), followed by OXA-48 (37%). The same study found considerable geographic differences in carbapenem-resistant *K. pneumoniae* (CRKP) distribution, with the highest rates in Southern and South-Eastern Europe, and also found differences in carbapenemase distribution between countries; in Romania, OXA-48-producing *K. pneumoniae* was the most frequently isolated carbapenemase (74%) [7]. The next major European study (carbapenem- and/or colistin-resistant *Enterobacteriaceae* survey) conducted by the European Centre for Disease Prevention and Control (ECDC) in 2019 found KPC to still be the most frequent carbapenemase in Europe (38.3%), followed by OXA-48 (28.9%) and NDM (15.3%) [8]. According to the European Antimicrobial Resistance Surveillance Network (EARS-Net) data, CRKP has been maintaining stable rates in Europe, ranging between 10% and 11.7% between 2020 and 2024, for invasive isolates [9]. The geographic disproportion in carbapenem resistance persisted over time, with data published by the EARS-Net for 2024 showing a range from 0% in Iceland to 67.6% in Bulgaria. In 2024, Romania displayed 50.3% carbapenem resistance for *K. pneumoniae* invasive isolates, being exceeded only by Bulgaria (67.6%) and Greece (60.2%), among the states that report data to the EARS-Net [10]. The first descriptions of carbapenemase-producing *K. pneumoniae* (CPKP) in Romania date back to 2010–2013, when OXA-48-producing and NDM-producing *K. pneumoniae* were reported in Central and Southern Romania [11,12]. Afterwards, the most frequent carbapenemase reported in Romania was OXA-48 [7] until 2022; since 2022–2023, the predominance of NDM+OXA-48-like-producing *K. pneumoniae* has been reported in Romania [13,14,15,16].

Most CPKP isolates are resistant to multiple classes of antibiotics. Among older antibiotics, tigecycline, fosfomycin, colistin and aminoglycosides still retain efficacy against some CRKP strains [8,17]. However, notable resistance to these antibiotics might be observed, likely due to their expanded use in regions where carbapenem-resistant Enterobacterales (CRE) infections were frequent, in the absence of other active antibiotics [18,19,20]. With the aim of treating CRGNB infections, new antibiotics have been approved over the last ten years, such as eravacycline, cefiderocol and novel beta-lactam/beta-lactamase inhibitors (i.e., ceftazidime–avibactam, meropenem–vaborbactam, imipenem–relebactam, and aztreonam–avibactam) [8]. Some of these agents have limited activity against CPKP, depending on the type of carbapenemase. Ceftazidime–avibactam can be used for both KPC and OXA-48-producing *K. pneumoniae* infections, whereas meropenem–vaborbactam and imipenem–relebactam are recommended only for KPC-producing *K. pneumoniae* infections [21]. Overall, MBLs are the most challenging carbapenemases, being susceptible only to aztreonam and cefiderocol, among beta-lactam antibiotics [8]. Aztreonam demands combination with avibactam in order to achieve activity against other beta-lactamases [22]. Thus, aztreonam–avibactam can be used for CPKP infections, regardless of the type of carbapenemase, including NDM+OXA-48-producing *K. pneumoniae*. This also applies to cefiderocol, albeit CPKP resistance to cefiderocol has already been described, most frequently among NDM-producing isolates [23,24].

Several studies reported high mortality rates in CRKP infections, with considerable variation from around 15% to over 60%, depending mainly on the type of infection [25,26,27,28,29]. Therefore, there is an increasing interest in researching different treatment strategies for CRKP infections, as well as defining indications for novel antibiotics, with most studies focusing on comparing the efficacy of antibiotic monotherapy with combination therapy. The findings of these studies are mainly controversial, reporting either the superiority of combination therapy [30,31] or similar outcomes between monotherapy and combination therapy [32,33,34].

Few studies address the recurrence of CRKP infections and their risk factors, and most concentrate only on bloodstream infections. In a study conducted between 2010 and 2016, Giannella et al. found high recurrence of CRKP bloodstream infections in patients receiving antibiotic regimens including colistin [35]. For CRE (predominantly *K. pneumoniae*), Alshehail et al. analyzed an extensive list of potential risk factors, of which only diabetes mellitus was shown to be associated with recurrence, the study being conducted on a number of 101 patients admitted between 2019 and 2024 [36].

Our study aims to describe the antibiotic resistance of CRKP in the post-COVID-19 pandemic period, analyze the correlation between antibiotic resistance and the types of carbapenemases produced by *K. pneumoniae*, assess the risk of recurrence for CRKP infections, and analyze the impact of adequate antibiotic treatment versus inadequate antibiotic treatment in CRKP infections.

## 2. Results

### 2.1. Antibiotic Resistance of K. pneumoniae

**Figure 1:**
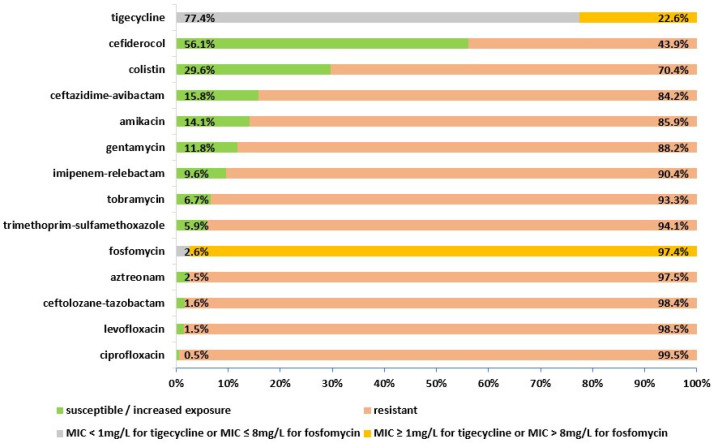
Carbapenem-resistant *K. pneumoniae* antibiotic susceptibility. MIC = minimum inhibitory concentration.

Two hundred and twenty-one CRKP isolates were collected from different types of samples: 17 blood cultures, 19 respiratory samples (sputum, tracheal aspirate, bronchial aspirate, and bronchoalveolar lavage), 159 urine cultures, and 26 samples of other origin. Seventeen of the 221 CRKP isolates were collected from nine other hospitals and sent to our medical facility for microbiological tests; these isolates were collected from blood cultures (four isolates), urine cultures (three isolates), respiratory samples (three isolates), and samples of other origin (seven isolates). The antibiotic susceptibility of the 221 CRKP isolates to routinely tested antibiotics is represented in Figure 1. Two hundred and ten CRKP isolates were carbapenemase producers: 116 (55.2%) NDM+OXA-48-producing *K. pneumoniae* isolates, 4 (1.9%) NDM+KPC-producing *K. pneumoniae* isolates, 55 (26.2%) NDM-producing *K. pneumoniae* isolates, 29 (13.8%) OXA-48-producing *K. pneumoniae* isolates, and 6 (2.9%) KPC-producing *K. pneumoniae* isolates. All except for six *K. pneumoniae* isolates (two NDM-producing isolates, two OXA-48-producing isolates, one KPC-producing isolate and one non-carbapenemase-producing isolate) were also extended-spectrum beta-lactamase (ESBL) producers. Antibiotic susceptibility and resistance profiles to routinely tested antibiotics, in accordance with the types of carbapenemases produced by *K. pneumoniae*, are displayed in Table S1.

Spearman’s rank correlation was used to measure the strength of the relationship between the types of carbapenemases and the antibiotic resistance to antibiotics displayed in Table S1. NDM-producing and NDM+OXA-48-producing *K. pneumoniae* were correlated with increased resistance to certain antibiotics:

- NDM+OXA-48-producing *K. pneumoniae* and aztreonam (100%, *p* = 0.01), ceftazidime–avibactam (100%, *p* < 0.01), trimethoprim–sulfamethoxazole (TMP-SMX) (99.1%, *p* < 0.01), gentamycin (94.8%, *p* < 0.01), amikacin (93.8%, *p* < 0.01), and colistin (79.8%, *p* < 0.01);
- NDM-producing *K. pneumoniae* and ceftazidime–avibactam (100%, *p* < 0.01).

KPC-producing, OXA-48-producing and non-carbapenemase-producing *K. pneumoniae* were correlated with decreased resistance to certain antibiotics:

- OXA-48-producing *K. pneumoniae* and ceftazidime–avibactam (11.5%, *p* < 0.01), amikacin (48.1%, *p* < 0.01), colistin (51.7%, *p* = 0.01), and gentamycin (65.5%, *p* < 0.01);
- KPC-producing *K. pneumoniae* and ceftazidime–avibactam (20%, *p* < 0.01), gentamycin (33.3%, *p* < 0.01), and TMP-SMX (50%, *p* < 0.01);
- non-carbapenemase-producing CRKP and ceftazidime–avibactam (44.4%, *p* < 0.01), ceftolozane–tazobactam (88.9%, *p* = 0.01), and levofloxacin (90%, *p* = 0.02).

Synergic effects of several combinations of antibiotics were tested for 51 isolates of CRKP (Table 1). These strains were isolated from blood cultures (four strains), urine cultures (41 strains), respiratory samples (four strains), and other sites (two strains). In vitro synergistic effects were found for 32/32 CRKP isolates tested to ceftazidime/avibactam plus aztreonam, 12/14 CRKP isolates tested to colistin plus tigecycline, and 2/11 CRKP isolates tested to antibiotic combinations including meropenem.

### 2.2. Characteristics of the Study Group and Assessment of the Risk of Recurrence for CRKP Infections

One hundred and sixty-six patients were included in the study. The differences between the control group (patients with a single hospital admission) and the case group (patients with at least two hospital admissions), regarding demographic, epidemiological and clinical characteristics, are displayed in Table 2. Patients’ ages ranged from 24 years to 92 years, with a median of 70 years (IQR: 60.75–74). No significant difference regarding age was found between the control group and the case group (median: 69.5 years vs. 70.5 years; Mann–Whitney U Test; *p* = 0.85). No significant difference was found between the case group and the control group regarding patients’ chronic diseases, represented by cardiovascular diseases (*p* = 0.53), neurological diseases (*p* = 0.19), nephrological diseases (*p* = 0.24), pulmonary diseases (*p* = 0.24), malignancy (*p* = 0.2), diabetes mellitus (*p* = 0.53) and obesity (*p* = 0.24). The patients in the case group, compared to the patients in the control group, more frequently accessed medical assistance for urinary tract conditions (96.2% vs. 63.6%; RR = 11.58, 95%CI: 1.58–81.91). The patients in the case group were more likely to present with double-J stents (DJS) or percutaneous nephrostomy (PCN) compared to the patients in the control group (23% vs. 5%; RR = 3.53; 95%CI: 1.72–7.22). During the 204 hospital visits of the 166 patients, 151 (74%) inpatients and 53 (26%) outpatients were recorded. The patients in the case group were more likely to be registered as outpatients than the patients in the control group (40.6% vs. 19.3%, χ^2^ = 10.4, *p* < 0.01). The median length of hospitalization for inpatients included in the whole group was 14 days (IQR: 9–25).

### 2.3. Antibiotic Treatment and Outcomes in Patients with CRKP Infections

**Figure 2:**
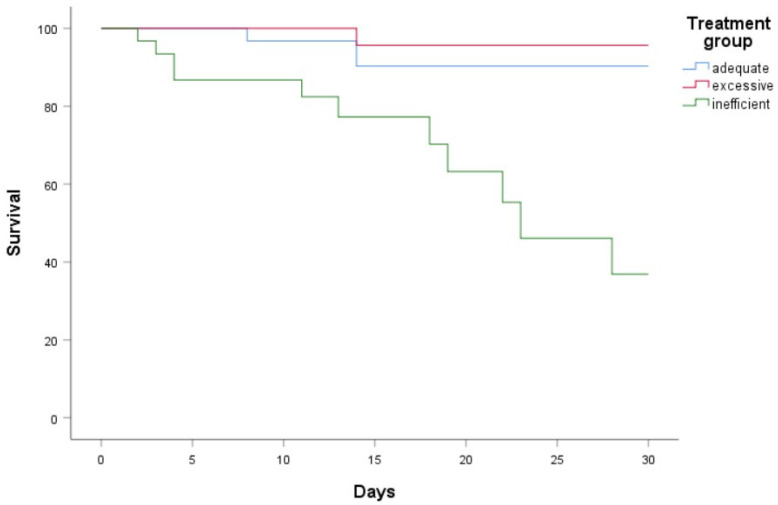
All-cause in-hospital 30-day mortality according to treatment groups in patients with CRKP infections or asymptomatic bacteriuria who were about to have an immediate urologic intervention. CRKP = carbapenem-resistant *K. pneumoniae*.

The patients included in the study received various antibiotic treatment regimens during 115 hospital admissions, represented by CRKP infections or asymptomatic bacteriuria in patients who were about to have an immediate urologic intervention in 101 cases, and by colonization in 14 cases. Within the group of 101 patients with CRKP infections or asymptomatic bacteriuria who were about to have an immediate urologic intervention, only 34 received adequate antibiotic treatment, 36 received excessive antibiotic treatment and 31 received inefficient antibiotic treatment. Among these 101 patients, all-cause in-hospital 30-day mortality was 13.8% (14/101). We evaluated the impact of adequate antibiotic treatment for CRKP infections on patients’ outcomes, compared to inadequate antibiotic treatment, either excessive or inefficient. The Kaplan–Meier curve of survival for these three groups is represented in Figure 2. Regarding all-cause in-hospital 30-day mortality, there was no significant difference between adequate and excessive antibiotic treatment (Log Rank Test; *p* = 0.38); the Log Rank Test indicated an increased all-cause in-hospital 30-day mortality in patients with inefficient antibiotic treatment (11/31), compared to both adequate (2/34; *p* = 0.01) and excessive antibiotic treatment (1/36; *p* < 0.01). In patients who received adequate antibiotic treatment, compared to patients who received excessive antibiotic treatment, we registered shorter treatment duration (median: 8 days vs. 12 days; Mann–Whitney U Test; *p* = 0.02) and shorter length of hospitalization (median: 13 days vs. 16 days; Mann–Whitney U Test; *p* = 0.04).

In the cases with CRKP isolates for which synergistic activity between specific antibiotics was found, patients received the specific combinations of antibiotics as follows: ceftazidime–avibactam and aztreonam (14 patients), colistin and tigecycline (six patients), meropenem and colistin (two patients), and tigecycline and fosfomycin (one patient). Table 3 displays the use of these combinations in the study group and the patients’ outcomes in cases where an infection was confirmed.

## 3. Discussion

Among the EU/EEA countries, Romania has a high prevalence of invasive infections produced by CRKP; the CRKP rates displayed an increasing trend in Romania, from 29.5% to 52.3% between 2018 and 2023, in spite of the less rapidly increasing population-weighted mean rates of CRKP in EU/EEA (7.5% in 2018 vs. 11.5% in 2023) [9,37,38]. Moreover, the estimated incidence (per 100,000 inhabitants) of CRKP bloodstream infections in Romania has reached first place in the EU/EEA in 2024; Romania had an estimated incidence of 20.31 in 2024, over five-fold higher than the average level reported by the EU/EEA [9]. In our medical facility, there was 21.4% carbapenem resistance in *K. pneumoniae* over six months of 2014 [39] and a similar rate of CRKP of 21.9% in 2019 [36]; afterwards, a rapidly increasing rate of CRKP was observed during the COVID-19 pandemic, to 34.1% in 2020 and 63.3% in 2021 [40].

A contributing factor for the increase in carbapenem resistance in *K. pneumoniae* is the high level of carbapenem consumption. Romania presented elevated and increasing carbapenem consumption, from 0.062 defined daily doses (DDD) per 1000 inhabitants per day in 2018 to 0.093 DDD per 1000 inhabitants per day in 2023, with a maximum of 0.114 DDD per 1000 inhabitants per day in 2021 during the COVID-19 pandemic. These levels continuously exceeded the mean of the EU/EEA carbapenem consumption, reaching a maximum of 2.28-fold in 2021, providing constant support for maintaining high levels of carbapenem resistance in Romania [38].

Carbapenemase production is known as the most common mechanism of carbapenem resistance in *K. pneumoniae*, with variations in the types of carbapenemases mainly according to geographic areas [7,8]. While the most frequently reported carbapenemase in the EU/EEA has constantly been KPC until now [7,8], Romania displayed changes in the prevalence of carbapenemases produced by *K. pneumoniae* over time. Previous data published by the authors have shown an OXA-48 predominance of 71.6% in CPE (mainly represented by *K. pneumoniae*) in 2014–2015 in our medical facility [41], in accordance with other studies from Romania, from the same period of time, which reported rates between 74% and 79% OXA-48-producing *K. pneumoniae* [3,42]. Afterwards, the rates of MBL-producing *K. pneumoniae* displayed a rapid increase from 3.8% to 41.6% between 2019 and 2021 in our medical facility [40]. The emergence of MBL-producing *K. pneumoniae* since 2021–2022 has also been notified by other studies conducted in Romania. These studies not only displayed the prevalence of NDM-producing *K. pneumoniae*, but also reported its predominant association with OXA-48, recording rates between 44.4% and 62.7% for NDM+OXA-48 association in CPKP isolates [13,14,15,16]. Our study presents comparable data, with 55.2% NDM+OXA-48-producing *K. pneumoniae*. The second most frequent CPKP was NDM-producing *K. pneumoniae* (26.2%), unlike the studies mentioned above, which reported OXA-48 as the second most frequent carbapenemase in *K. pneumoniae* (between 9.3% and 35.5%) [13,14,15,16]. This suggests a high possibility that NDM-producing *K. pneumoniae*, with or without OXA-48, is becoming the main resistance mechanism to be taken into account when managing a CRKP infection. The predominant coharboring of NDM and OXA-48 in *K. pneumoniae*, scarcely reported in Romania before 2020 and widely expanded afterwards, is massively increasing the burden of *K. pneumoniae* infections, as these strains exhibit high rates of resistance to many antibiotics [13,14,15,16].

In our study, NDM+OXA-48-producing *K. pneumoniae* was correlated with increased resistance to colistin (79.8%), TMP-SMX (99.1%) and aminoglycosides—amikacin (93.8%) and gentamycin (94.8%). This phenomenon of increased resistance has been previously reported in NDM+OXA-48-producing *K. pneumoniae*, highlighting the loss of effectiveness of last-resort antibiotics in the treatment of CPKP infections. A study conducted in Bucharest, Romania, by Lazar et al., reported susceptibility below 10% to aminoglycosides and TMP-SMX and 33% susceptibility to colistin for NDM+OXA-48-producing *K. pneumoniae* in 2022–2023 [13]; another study, conducted by Melinte et al., also in Bucharest, Romania, reported 100% resistance to amikacin and TMP-SMX and 58.3% colistin resistance in NDM- or NDM+OXA-48-producing *K. pneumoniae* in 2023 [16]. In our study, there were significant correlations between OXA-48-producing *K. pneumoniae* and lower resistance rates to colistin (51.7%) and aminoglycosides—amikacin (48.1%) and gentamycin (65.5%)—as well as KPC-producing *K. pneumoniae* and lower resistance rates to gentamycin (33.3%) and TMP-SMX (50%). The study conducted by Melinte et al., concerning antibiotic resistance rates of different types of CPKP, showed similar differences for NDM+OXA-48 compared to OXA-48- or KPC-producing *K. pneumoniae*, with 60% TMP-SMX resistance in KPC-producing *K. pneumoniae*, as well as 31.3% amikacin resistance, 75% colistin resistance, and 81.3% TMP-SMX resistance in OXA-48-producing *K. pneumoniae* [16]. Another study conducted by Lazar et al. reported decreasing colistin susceptibility in NDM- or VIM-producing *K. pneumoniae* from 2023 to 2024 (50% vs. 41.4%), while in OXA-48- or KPC-producing *K. pneumoniae*, the colistin susceptibility increased from 48% to 62% from 2023 to 2024 [14]. Therefore, colistin, TMP-SMX and aminoglycosides might still be valid therapeutic options in some infections produced by OXA-48- or KPC-producing *K. pneumoniae*. Nevertheless, according to the highly increasing prevalence of NDM- or NDM+OXA-48-producing *K. pneumoniae*, the utility of these antibiotics is diminishing, frequently requiring the use of cefiderocol, aztreonam–avibactam, or other active synergistic antibiotic combinations in the management of CPKP infections. However, resistance to aminoglycosides, colistin and TMP-SMX is not determined by carbapenemase production, but only statistically correlated with different types of carbapenemases; a possible reason for these correlations might be represented by a linked transmission of resistance to carbapenems and other classes of antibiotics on the same mobile genetic elements.

In our study several isolates of *K. pneumoniae* required antibiotic synergy testing because of the lack of treatment options, either due to the lack of effectiveness or because of the adverse effects produced by the in vitro active antibiotics. All the CRKP isolates tested for ceftazidime–avibactam plus aztreonam were susceptible to this combination. The combination of ceftazidime–avibactam and aztreonam, which mediates aztreonam association with avibactam, is well known to be active against all types of CPKP, including isolates coharboring NDM and OXA-48 [22]. Aztreonam–avibactam is currently approved for clinical use in Romania for infections caused by Gram-negative bacteria (including *K. pneumoniae*); however, in order to avoid its rapid dismantlement of effectiveness, its usage is recommended to be restricted only for cases without other treatment options, after a prior advisory with an infectious diseases physician experienced in managing antibiotic resistance [43]; the same practice is recommended for the use of all the novel antibiotics—ceftazidime–avibactam [44], imipenem–relebactam [45], meropenem–vaborbactam [46], and cefiderocol [47]. Synergic effects for several other antibiotic combinations were found in our study for a limited number of *K. pneumoniae* isolates (between one and 12 isolates); these combinations were represented by colistin plus tigecycline, colistin plus fosfomycin, colistin plus gentamycin, colistin plus amikacin, colistin plus meropenem, tigecycline plus fosfomycin, and tigecycline plus amikacin. When testing antibiotic combination regimens that include meropenem, the minimum inhibitory concentration (MIC) of meropenem should be below 16 mg/dL [48,49]. In our study, there were no antagonistic effects found for the tested antibiotic combinations. Investigating potential synergistic effects between older antibiotics is a key approach to finding treatment solutions for CRKP infections. Several published studies found effective antibiotic synergy between some combinations of antibiotics, such as tigecycline and colistin [50,51], fosfomycin and meropenem [52,53], fosfomycin and colistin [52,53], colistin and meropenem [54], and fosfomycin and amikacin [55]. However, these studies are conducted on a limited number of *K. pneumoniae* isolates, reporting in vitro results, without offering a broad overview for all carbapenemases; therefore, they do not yet provide a clear and reliable image of the future perspective for the treatment of CRKP infections.

Several patients registered multiple hospital admissions for recurrent CRKP infections in our study; the recurrence of CRKP infections was recorded significantly more often in patients addressing medical assistance for urinary tract conditions, with higher significance in patients with upper urinary tract devices (PCN or DJS). The association with upper urinary tract devices is probably supported by biofilm formation, which establishes a protective environment for *K. pneumoniae*, providing a basis for relapsing urinary tract infections, which become difficult to treat [56]. Therefore, we consider that retaining an upper urinary tract device for longer than it is required is not recommended and that the prompt removal whenever it is possible might reduce the burden of CRKP infections. Furthermore, no other risk factor for the recurrence of CRKP infections, within either demographic or epidemiological features, could be found in our study. Our data also showed that patients’ comorbidities were not associated with the recurrence of CRKP infections. However, a study conducted by Alshehail et al. reported diabetes mellitus as a risk factor for the recurrence of CRE (mainly *K. pneumoniae*) infections [36]. In our study, diabetes mellitus was not associated with the recurrence of CRKP infections, but the lack of association might be due to the small number of patients with recurrences.

Regarding patients’ outcomes, a 13.8% all-cause in-hospital 30-day mortality rate was registered in our study in CRKP infections or asymptomatic bacteriuria in patients who were about to have an immediate urologic intervention. Considering the high resistance rates of CRKP to almost all the tested antibiotics, we aimed to analyze whether adequate treatment of CRKP infections displayed significant differences compared to excessive or inefficient antibiotic treatment. Thus, as expected, in patients receiving inefficient antibiotic treatment, the survival rate was lower than in both patients receiving adequate or excessive antibiotic treatment; no significant difference was found between adequate and excessive treatment in terms of survival. Nevertheless, adequate antibiotic treatment of CRKP infections was associated with both shorter treatment duration and shorter length of hospitalization compared to excessive antibiotic treatment; these associations point out that the selection of adequate antibiotic treatment not only spare unnecessary antibiotics, but also reduce patients’ exposure to complications determined by prolonged antibiotic treatment (additional adverse effects and higher risk of developing supplementary antibiotic resistance) [57] and hospitalization (including hospital-acquired infections, depression, physical deconditioning, and even death) [58,59]. Published studies concerning the treatment of CRKP infections mainly focus on comparing antibiotic monotherapy with combination therapy and are less related to the comparison of adequate antibiotic treatment with inadequate antibiotic treatment [30,31,32,33,34,60]. A literature review, published in 2014 by Tzouvelekis et al., reported similar mortality rates between CPKP infections receiving monotherapy (colistin, tigecycline or carbapenems) and CPKP infections receiving inadequate antibiotic therapy (38.7% vs. 46.1%), but found combination therapy (mortality rate of 27.4%) to be superior to monotherapy [30]. Another study, published in 2017, described a significantly lower mortality rate for CRKP infection receiving monotherapy (colistin, tigecycline or carbapenems) compared to inadequate treatment (21.3% vs. 37.5%) [60]. More recent studies on ceftazidime–avibactam have shown similar outcomes for its monotherapy compared with its combination therapy in infections produced by CRGNB, especially KPC-producing *K. pneumoniae* [32,33]. Cefiderocol monotherapy, compared to other best available antibiotic treatments, appears to have similar clinical effect and microbiological efficacy in CRGNB (*K. pneumoniae*, *Acinetobacter baumannii*, and *Pseudomonas aeruginosa*) infections [34]. However, a systematic review and meta-analysis on the efficacy of combination therapy versus monotherapy in the treatment of CRGNB infections, published in 2024, stated that monotherapy had higher mortality rates and lower microbiological eradication compared to combination therapy in CRE infections [31].

In our study, in a limited number of 21 cases of CRKP infections, four antibiotic combinations with effective in vitro synergy were used (ceftazidime–avibactam plus aztreonam, tigecycline plus colistin, meropenem plus colistin, and tigecycline plus fosfomycin). Two deaths were recorded among the 21 patients, implying that in all the other 19 cases, the antibiotic combinations were successfully used. The successful usage of these combinations in the majority of cases suggests that they may be a treatment option for CRKP infections in those situations in which novel active antibiotics are not available. Further studies, on a larger scale and including control groups, are needed to confirm the effectiveness of these antibiotic combinations.

### Study Limitations

Our study has several limitations. The first limitation is the retrospective nature of the study; therefore, some of the demographic, epidemiological and clinical data might not have been available in the patients’ clinical records. The second limitation is that the study was conducted in a hospital strictly dedicated to the management of patients with infectious diseases, which means that its findings might not be applicable in other medical facilities with extended activity in other medical specialties. Afterwards, the small number of patients that were acquired in the study groups after the divisions of the whole lot may diminish the strength of the statistical analysis; the same statement applies to treatment analysis because of the high variability of treatment regimens, the small number of patients receiving the same treatment regimen and the variety of carbapenemases produced by *K. pneumoniae*. The antibiotic treatment was analyzed only for CRKP infections or colonization with antibiotic susceptibility testing (AST) results; therefore, the initial antibiotic treatment is not addressed in this study.

## 4. Materials and Methods

We conducted a retrospective, cross-sectional study that included all the patients with CRKP infections or colonization admitted to the National Institute of Infectious Diseases “Prof. Dr. Matei Balș” (NIIDMB) between January 2024 and December 2024. Inclusion criteria were represented by:

- Patients with a confirmed infection or colonization determined by CRKP (where CRKP was represented by *K. pneumoniae* resistant to at least one carbapenem, either imipenem, meropenem or ertapenem, according to the European Committee on Antimicrobial Susceptibility Testing (EUCAST) breakpoints version 14.0, applicable in 2024) [61].
- Each individual infectious event produced by CRKP was included in the study (in cases where a patient recorded multiple hospital visits for successive infectious events).

Exclusion criteria were represented by:

- In order to eliminate the duplicates, only one isolate of CRKP per hospital visit was included in the study, selected by the following criteria:
  ○ If CRKP was isolated from blood cultures, the first blood culture was included in the study.
  ○ In all the other cases, the first isolate of CRKP collected during admission was included in the study.
- Patients with rectal carriage of CRKP.

### 4.1. Characteristics of the Study Group

**Figure 3:**
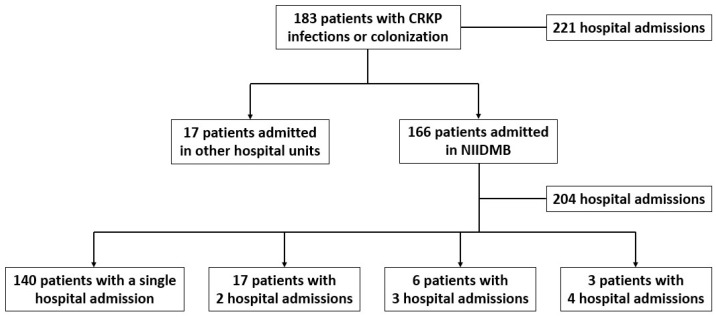
Patients with CRKP infections/colonization included in the study group. CRKP = carbapenem-resistant *K. pneumoniae.* NIIDMB = National Institute of Infectious Diseases “Prof. Dr. Matei Balș”.

A total of 183 patients were included in the study, which recorded 221 hospital admissions. Seventeen of the 183 patients were admitted to other hospital units, and only microbiological tests were performed in NIIDMB for these patients, for which we only had information about the AST of CRKP, without access to any clinical or epidemiological data. Each of the remaining 166 patients included in the study group recorded between one and four hospital visits for infectious events (Figure 3).

Within the antibiotic resistance analysis, we included all 221 CRKP isolates collected from the patients included in the study group. Within the demographic, epidemiological, and clinical analysis, we included the 166 patients admitted to NIIDMB.

The epidemiological and clinical data were collected from the hospital electronic database. The analysis included the following parameters and variables: patients’ age and sex, patients’ comorbidities, type of hospital visit (inpatients or outpatients), length of hospital stay, type of sample from which CRKP was isolated, clinical significance for the presence of CRKP (infection or colonization), site of infection (bloodstream infection, urinary tract infection, pneumonia, skin and soft tissue infection, infections with other sites, or septic shock), antibiotic treatment, presence of medical devices, and outcome (all-cause in-hospital 30-day mortality).

We divided the 166 patients into two groups, the control group (patients with a single hospital admission—140 patients) and the case group (patients with at least two hospital admissions—26 patients), in order to assess the risk of recurrence for CRKP infections. Prior hospitalization was represented by hospitalization in the last three months before admission.

### 4.2. Definitions

Antibiotic treatment for CRKP infections was divided into appropriate or inappropriate treatment:

- Appropriate treatment was defined as treatment with an active antibiotic against the CRKP isolate, if the antibiotic was used in the right dose, if the antibiotic was able to reach the infected tissue and if it was the one with the narrowest spectrum from the available treatment options for the CRKP infection.
- Inappropriate treatment was defined as:
  ○ Excessive, if broader spectrum antibiotic agents (including antibiotic associations) were used when a narrower spectrum antibiotic was advisable.
  ○ Inefficient, when the antibiotic was not active against the CRKP isolate.

All cases of colonization that received antibiotic treatment were classified as inappropriate, with the exception of asymptomatic bacteriuria in patients who were about to have an immediate urologic intervention; for these patients, antibiotic treatment was defined in the same way as for infections.

### 4.3. Isolation and Antibiotic Susceptibility Testing

*K. pneumoniae* isolates were identified using matrix-assisted laser desorption ionization time-of-flight mass spectrometry (MALDI-TOF MS) Biotyper (Bruker Daltonics GmbH & Co., KG, Bremen, Germany), with bacterial spectra being analyzed using the Biotyper software v. 3.1. Antibiotic susceptibility was tested using automatic systems (MICRONAUT—Bruker Daltonics GmbH & Co., KG, Bremen, Germany). The antibiotics accounting for the routine AST were amoxicillin–clavulanic acid, ampicillin–sulbactam, piperacillin–tazobactam, cefoxitin, ceftriaxone, cefotaxime, ceftazidime, cefepime, ceftazidime–avibactam, ceftolozane–tazobactam, aztreonam, ertapenem, imipenem, meropenem, imipenem–relebactam, amikacin, gentamycin, tobramycin, ciprofloxacin, levofloxacin, colistin, fosfomycin, trimethoprim–sulfamethoxazole, tigecycline, and cefiderocol. The antibiotic resistance breakpoints were used according to the EUCAST breakpoints version 14.0, applicable in 2024, for interpretation of susceptibility testing [61]. For tigecycline, the PK-PD breakpoint of MIC ≥ 1 mg/L was used for establishing resistance to high-dose tigecycline (EUCAST recommendation) [62]. For fosfomycin, MIC > 8 mg/L was used for establishing resistance to fosfomycin (EUCAST recommendation) [63].

Carbapenem resistance was defined as resistance to at least one carbapenem, either ertapenem, imipenem, or meropenem. All the strains with MIC for meropenem of over 0.125 mg/L were tested for ESBL and carbapenemase production according to the EUCAST guideline for the detection of resistance mechanisms v2.0 (2017). ESBL production was assessed using lateral flow assays—NG Test/CTX-M Multi (NG Biotech, Guipry-Messac, France). The types of carbapenemases were identified using a phenotypic method: immunochromatographic lateral flow assay from Coris BioConcept, Gembloux, Belgium (Resist-3 O.K.N. *K*-SeT) to detect OXA-48 type, KPC, and NDM carbapenemases [64].

Synergic activity testing of several antibiotic combinations was analyzed in 51 CRKP isolates for the following antibiotics: ceftazidime–avibactam + aztreonam, meropenem + colistin, colistin + amikacin, colistin + gentamycin, i.v. fosfomycin + tigecycline, tigecycline + colistin, tigecycline + amikacin, colistin + i.v. fosfomycin, meropenem + tigecycline, meropenem + levofloxacin, and amikacin + i.v. fosfomycin. Synergic activity testing was performed for extensively drug-resistant or pan drug-resistant CRKP isolates, in cases of patients with:

- CRKP infections when CRKP isolates were susceptible to only one antibiotic which cannot be used as monotherapy;
- Urinary tract infections with CRKP isolates with MIC < 1 mL/L for tigecycline and resistance to other available antibiotics;
- Kidney disease with infections produced by CRKP isolates susceptible to only one or two nephrotoxic antibiotics.

The synergic activity analysis was performed using the *E*-test synergy testing (MIC/MIC ratio) methodology (AB Biodisk, Solna, Sweden). First, the strain was tested for each antibiotic susceptibility intended to be used in combination using the *E*-test (gradient diffusion strip) in order to find the exact MIC. Then, one of the antibiotics (A) tested for synergy was placed on a Mueller–Hinton agar plate, previously inoculated with 0.5 McFarland concentration of the strain, for 1 h at room temperature, and the MIC for antibiotic A was marked on the back of the plate. Afterwards, antibiotic A was removed and the other antibiotic (B) was placed in place of antibiotic A in such a way that the MIC of antibiotic B overlapped the MIC of antibiotic A. The plate was then placed into the incubator and read after 24 h. The respective MIC strips were then used to read the combination MIC AB. The Fractional Inhibitory Concentration Index (FIC Index) was calculated and the antibiotic synergy of combination agents was interpreted as follows: synergy (MIC of combination is ≥2 dilutions lower than MIC of the most active drug alone), antagonism (MIC of combination is ≥2 dilutions higher than MIC of the most active drug alone) and indifference (MIC of combination is within ±1 dilution compared to the most active drug alone).

### 4.4. Statistical Analysis

Data were collected from the electronic database of the hospital using Microsoft Office 2019 software. We used SPSS Statistics 26 software and MedCalc 23.5.5 statistical software for data analysis: Kolmogorov–Smirnov Test for the assessment of normal distribution, Mann–Whitney U Test for continuous variables with non-normal distribution, relative risk, Z-score for proportions, Chi-Square test and Spearman’s Rho Correlation for categorical variables, and Kaplan–Meier curve for survival analysis. The results were considered to be statistically significant at a *p*-value < 0.05, measured by two-tailed tests.

## 5. Conclusions

Producing NDM+OXA-48 carbapenemases was the main antibiotic resistance mechanism of *K. pneumoniae* identified in our study. This association of carbapenemases was correlated with increased resistance to multiple antibiotics, such as trimethoprim–sulfamethoxazole, gentamycin, amikacin and colistin, indicating diminished activity of many antibiotics in the management of CRKP infections. In vitro synergistic activity was noted in all the CRKP isolates tested to ceftazidime/avibactam plus aztreonam and in most CRKP isolates tested to colistin plus tigecycline, providing an argument for using these combinations in CRKP infections for which there are no other available treatment options.

Based on the results of our study, urinary tract conditions, particularly those accompanied by upper urinary tract devices, are associated with a higher risk of recurrence of CRKP infections, indicating that removing the devices as soon as possible is advisable in order to reduce the potential burden of CRKP infections. We found adequate antibiotic treatment to be superior to excessive antibiotic treatment in CRKP infections due to the fact that adequate antibiotic treatment was associated with shorter treatment duration and shorter length of hospitalization, thus potentially being able to reduce patients’ complications determined by prolonged antibiotic treatment or hospitalization. Nevertheless, a high rate of excessive antibiotic treatment was recorded in our study; this finding points out the need for greater attention when choosing an antibiotic regimen and for improved medical training.

Considering the high levels of antibiotic resistance of CRKP to many antibiotics and the high rates of MBL-producing *K. pneumoniae*, investigating future treatment strategies and improving rapid carbapenemase detection, alongside prudent antibiotic consumption and implementing wide infection prevention and control measures, are essential for the future approach to these CRKP infections. Moreover, microbiological results, including the characterization of the types of carbapenemases, should always be taken into account if they are available when the antibiotic regimen is being prescribed. Additionally, we consider that active antibiotic combinations are advisable to be used whenever possible in order to spare carbapenem consumption, which only amplifies the selection of CRKP.

## Figures and Tables

**Table 1:** Antibiotic synergism against carbapenem-resistant *K. pneumoniae*.

Antibiotic Association	Tested Isolates (N)	Synergy (N)	Indifference (N)	Antagonism (N)
Ceftazidime/avibactam—Aztreonam	32 strains	32 strains	None	None
Colistin—Tigecycline	14 strains	12 strains	2 strains	None
Colistin—Fosfomycin	9 strains	2 strains	7 strains	None
Colistin—Meropenem	7 strains	2 strains	5 strains	None
Tigecycline—Fosfomycin	6 strains	2 strains	4 strains	None
Tigecycline—Meropenem	3 strains	None	3 strains	None
Colistin—Amikacin	2 strains	1 strain	1 strain	None
Fosfomycin—Amikacin	1 strain	None	1 strain	None
Levofloxacin—Meropenem	1 strain	None	1 strain	None
Colistin—Gentamycin	1 strain	1 strain	None	None
Tigecycline—Amikacin	1 strain	1 strain	None	None

Synergy = MIC of the combination is ≥2 dilutions lower than the MIC of the most active drug alone. Antagonism = MIC of the combination is ≥2 dilutions higher than the MIC of the most active drug alone. Indifference = MIC of the combination is within ±1 dilution compared to the most active drug alone. MIC = minimum inhibitory concentration.

**Table 2:** Demographic, epidemiological and clinical characteristics of patients included in the study.

Variable	Whole Study Group (166 Patients)	Study Groups in accordance with the Number of Hospital Admissions			
		Control Group (140 Patients)	Case Group (26 Patients)	RR (95% CI)	z-Score, *p*-Value
Demographics and epidemiology					
Male (N, %)	112 (67.4%)	92 (65.7%)	20 (76.9%)	1.6 (0.68; 3.76)	z = 1.09, *p* = 0.27
Prior hospitalization (N, %)	103 (62%)	89 (63.6%)	14 (53.8%)	0.71 (0.35; 1.44)	z = 0.93, *p* = 0.34
Long-term care facility (N, %)	14 (8.4%)	14 (10%)	0 (0%)	0.19 (0.01; 3)	z = 1.17, *p* = 0.23
Inter-hospital transfer (N, %)	39 (23.4%)	39 (27.9%)	0 (0%)	0.06 (0; 0.96)	z = 1.98, *p* = 0.04
Medical devices					
Endotracheal intubation and mechanical ventilation (N, %)	30 (18%)	30 (21.4%)	0 (0%)	0.08 (0; 1.33)	z = 1.75, *p* = 0.07
Central venous catheter (N, %)	35 (21%)	35 (25%)	0 (0%)	0.06 (0; 1.1)	z = 1.88, *p* = 0.05
Nasogastric tube (N, %)	32 (19.2%)	32 (22.9%)	0 (0%)	0.07 (0; 1.23)	z = 1.81, *p* = 0.07
PCN or DJS (N, %)	13 (7.8%)	7 (5%)	6 (23%)	3.53 (1.72; 7.22)	z = 3.45, *p* < 0.01
Infection site					
Bloodstream infection (N, %)	2 (1.2%)	2 (1.4%)	0 (0%)	1.03 (0.08; 13.34)	z = 0.02, *p* = 0.97
Respiratory site (N, %)	7 (4.2%)	7 (5%)	0 (0%)	0.37 (0.02; 5.65)	z = 0.7, *p* = 0.48
SSTI (N, %)	8 (4.8%)	8 (5.7%)	0 (0%)	0.33 (0.02; 5.04)	z = 0.79, *p* = 0.42
Septic shock (N, %)	18 (10.8%)	18 (12.9%)	0 (0%)	0.14 (0; 2.33)	z = 1.35, *p* = 0.17
Urinary site (either infection or asymptomatic bacteriuria) (N, %)	114 (86.7%)	89 (63.6%)	25 (96.2%)	11.58 (1.58; 81.91)	z = 2.41, *p* = 0.01
Other site colonization (N, %)	10 (6%)	10 (7.1%)	0 (0%)	0.26 (0.01; 4.13)	z = 0.94, *p* = 0.34
Infections with other sites (N, %)	2 (1.2%)	1 (1.4%)	1 (3.8%)	2.17 (0.42; 11.2)	z = 0.92, *p* = 0.35
Undetermined infection or colonization (N, %)	4 (2.4%)	4 (2.9%)	0 (0%)	0.61 (0.04; 8.71)	z = 0.35, *p* = 0.71

DJS = double-J stent. PCN = percutaneous nephrostomy. SSTI = skin and soft tissue infection.

**Table 3:** Antibiotic treatment options and outcomes in patients where synergistic activity was found.

Antibiotic Treatment		Infections				Colonization
		Total Number of Patients (N)	Outcome			Total Number of Patients (N)
			Recovery (N)	Recurrence (N)	Death (N)	
Aztreonam + ceftazidime/avibactam	Without other antibiotics ^1^	9	4	5	-----	-----
	Preceded or succeeded by other antibiotic schemes ^2^	5	3	1	1	-----
Tigecycline + colistin	Without other antibiotics ^1^	2	2	-----	-----	-----
	Preceded or succeeded by other antibiotic schemes ^2^	3	2	-----	1	1
Meropenem + colistin	Without other antibiotics ^1^	-----	-----	-----	-----	-----
	Preceded or succeeded by other antibiotic schemes ^2^	1	1	-----	-----	1
Tigecycline + fosfomycin	Without other antibiotics ^1^	1	1	-----	-----	-----
	Preceded or succeeded by other antibiotic schemes ^2^	-----	-----	-----	-----	-----
Total		21	13	6	2	2

^1^ Patients did not receive any other antibiotic treatment than the specific combination of antibiotics as part of treatment for the CRKP infection. ^2^ Patients received the specific combination of antibiotics, preceded or succeeded by other antibiotics, as part of treatment for the CRKP infection.

## Data Availability

The raw data supporting the conclusions of this article will be made available by the authors upon request.

## Supplementary Materials

The following supporting information can be downloaded at https://www.mdpi.com/article/10.3390/antibiotics15060533/s1. Table S1: Antibiotic resistance of CPKP in accordance with the carbapenemase-producing profile.

## Abbreviations

The following abbreviations are used in this manuscript:

AST	antibiotic susceptibility testing
CPKP	carbapenemase-producing *K. pneumoniae*
CRE	carbapenem-resistant Enterobacterales
CRGNB	carbapenem-resistant Gram-negative bacteria
CRKP	carbapenem-resistant *K. pneumoniae*
DDD	defined daily doses
DJS	double-J stents
EARS-Net	European Antimicrobial Resistance Surveillance Network
ECDC	European Centre for Disease Prevention and Control
ESBL	extended-spectrum beta-lactamase
EUCAST	European Committee on Antimicrobial Susceptibility Testing
EuSCAPE	European Survey on Carbapenemase-Producing *Enterobacteriaceae*
IMP	imipenemase
KPC	*K. pneumoniae* carbapenemase
MBL	metallo-beta-lactamases
MIC	minimum inhibitory concentration
NDM	New Delhi metallo-beta-lactamase
NIIDMB	National Institute of Infectious Diseases “Prof. Dr. Matei Balș”
OXA	oxacillinase
PCN	percutaneous nephrostomy
SSTI	skin and soft tissue infection
TMP-SMX	trimethoprim–sulfamethoxazole
VIM	Verona integron-encoded metallo-beta-lactamase
