# Supplementary material for: Carbapenem-Resistant Klebsiella pneumoniae: Carbapenemase Production, Antibiotic Resistance and Treatment Options, in an Infectious Diseases Hospital from Romania
Source: Antibiotics (Basel). 2026 May 24;15(6):533. doi: 10.3390/antibiotics15060533 (PMC13296309; doi:10.3390/antibiotics15060533)
Supplement: Supplementary file 1 [file antibiotics-15-00533-s001.zip › antibiotics-4315473-supplementary.pdf]

Table S1. Antibiotic resistance of CPKP in accordance with the carbapenemase-producing profile.

Antibiotic		Non-carbapenemase-producing CRKP	Carbapenemase-producing *Klebsiella pneumoniae* isolates					Total
			NDM+OXA-48	NDM+KPC	NDM	OXA-48	KPC	
**Cefiderocol**	Tested (N)	5	90	4	42	12	2	155
	Resistant (N)	2	37	2	21	4	2	68
	Susceptible (N)	3	53	2	21	8	0	87
**Aztreonam**	Tested (N)	10	107	4	50	27	5	203
	Resistant (N)	9	107	4	49	24	5	198
	Susceptible (N)	1	0	0	1	3	0	5
**Ceftazidime-avibactam**	Tested (N)	9	107	4	51	26	5	202
	Resistant (N)	4	107	4	51	3	1	170
	Susceptible (N)	5	0	0	0	23	4	32
**Ceftolozane-tazobactam**	Tested (N)	9	105	4	49	22	4	193
	Resistant (N)	8	105	4	49	20	4	190
	Susceptible (N)	1	0	0	0	2	0	3
**Fosfomycin**	Tested (N)	9	90	2	32	18	3	154
	MIC > 8mg/L (N)	9	89	2	31	16	3	150
	MIC ≤ 8mg/L (N)	0	1	0	1	2	0	4
**Tobramycin**	Tested (N)	9	93	4	44	24	4	178
	Resistant (N)	9	88	4	41	20	4	166
	Susceptible (N)	0	5	0	3	4	0	12
**Gentamycin**	Tested (N)	11	115	4	55	29	6	220
	Resistant (N)	11	109	4	49	19	2	194
	Susceptible (N)	0	6	0	6	10	4	26
**Amikacin**	Tested (N)	11	113	4	52	27	6	213
	Resistant (N)	9	106	4	46	13	5	183
	Susceptible (N)	2	7	0	6	14	1	30
**Levofloxacin**	Tested (N)	10	103	4	49	26	4	196
	Resistant (N)	9	103	4	47	26	4	193
	Susceptible (N)	1	0	0	2	0	0	3
**Ciprofloxacin**	Tested (N)	10	110	4	53	28	6	211
	Resistant (N)	10	110	4	52	28	6	210
	Susceptible (N)	0	0	0	1	0	0	1
**Colistin**	Tested (N)	9	114	4	54	29	6	216
	Resistant (N)	6	91	2	34	15	4	152
	Susceptible (N)	3	23	2	20	14	2	64
**TMP-SMX**	Tested (N)	11	115	4	55	29	6	220
	Resistant (N)	9	114	4	51	26	3	207
	Susceptible (N)	2	1	0	4	3	3	13
**Tigecycline**	Tested (N)	5	84	3	40	12	2	146
	MIC ≥ 1mg/L (N)	2	21	1	6	3	0	33
	MIC < 1mg/L (N)	3	63	2	34	9	2	113

CPKP = carbapenemase-producing *Klebsiella pneumoniae*. MIC = minimum inhibitory concentration.
